# Time-restricted eating in adults with type 2 diabetes mellitus on concomitant glucagon-like peptide-1 receptor agonists: case report

**DOI:** 10.3389/fcdhc.2026.1725278

**Published:** 2026-06-23

**Authors:** Kyla L. Laing, Jordan L. Levy, Michael J. Wilkinson, Shweta Varshney, Satchidananda Panda, Pam R. Taub, Emily N. C. Manoogian

**Affiliations:** 1 Regulatory Biology, Salk Institute for Biological Studies, La Jolla, CA, United States; 2 Division of Cardiovascular Medicine, Department of Medicine, University of California, San Diego, San Diego, CA, United States

**Keywords:** case report, continuous glucose monitor, glucagon-like peptide-1 receptor agonist, lifestyle intervention, time-restricted eating, type 2 diabetes mellitus

## Abstract

Type 2 diabetes mellitus (T2DM), marked by insulin resistance and impaired blood glucose regulation, can lead to serious complications when poorly controlled. Recent studies highlight interactions between diet, sleep, and metabolic disorders, showing that elevated nighttime glucose levels are associated with a higher risk of cardiometabolic disorders. Time-restricted eating (TRE) is a lifestyle strategy that aligns dietary intake with the circadian system with a consistent 8- to 10-hour eating window during the day. TRE is a safe and effective lifestyle intervention that improves cardiovascular health and glycemic control, and may provide additional benefits beyond stable use of a glucagon-like peptide-1 receptor agonist (GLP-1RA). The three patients presented here participated in a clinical trial for adults with T2DM in which they were instructed to follow a consistent 8 to 10-hour TRE intervention for 3 months (NCT05365529). Assessments, including fasted blood draws, 10-day continuous glucose monitor (CGM) use, and 14-day actigraphy to measure sleep-wake cycles, were conducted at baseline and 3 months. At baseline, all 3 patients were on stable GLP-1RA and exhibited high mean glucose levels (>130mg/dL) during their nightly sleep. Following the 3-month TRE intervention, all 3 patients experienced reductions in hemoglobin A1c (HbA1c) and nighttime glucose, along with improved time in range (TIR) during sleep. This case series highlights the potential benefits of TRE, alongside GLP-1RA use, on nighttime glucose levels, while also underscoring the potential of CGM to identify glycemic phenotypes that may predict specific responses to TRE, demonstrating the importance of a personalized approach.

## 1 Introduction

Type 2 diabetes mellitus (T2DM) is a chronic metabolic disease characterized by insulin resistance and impaired blood glucose regulation, affecting an estimated 36.1-38.1 million people in the United States (1) and more than half a billion people worldwide (2). When poorly managed, T2DM can lead to dangerous micro- and macrovascular complications, including retinopathy, neuropathy, nephropathy, and atherosclerotic cardiovascular disease – the leading cause of mortality worldwide (3). In the United States, diabetes is the 8th leading cause of mortality and is associated with economic costs exceeding $400 billion. Despite substantial investments in public health initiatives and pharmacotherapy, the prevalence of T2DM is projected to continue rising, along with the economic burden it places on both individuals and the healthcare system (4). Traditional treatment approaches for T2DM, which include a combination of pharmacological and behavioral interventions (such as diet and exercise), have been insufficient. Glucagon-like peptide-1 receptor agonists (GLP-1RAs) may provide substantial benefits, including lowering hemoglobin A1c (HbA1c), promoting weight loss, and reducing cardiovascular (CV) risk; however, they are also associated with adverse effects, most commonly gastrointestinal symptoms (5). Time-restricted eating (TRE), which aligns dietary intake to 8 to 10 hours during the daytime active phase, presents a potential lifestyle intervention to improve glycemic control and decrease CV risk. A recent randomized controlled trial demonstrated that TRE lowered HbA1c and reduced intra- and inter-daily glycemic variability, both of which are linked to increased CV risk (6). Traditional methods for monitoring blood glucose levels, including fasting blood draws and finger sticks, provide an incomplete picture and do not offer insight into diurnal patterns of glucose levels. Continuous glucose monitors (CGM), which estimate blood glucose levels every 5–15 minutes via interstitial fluid for 10–14 days, present a novel tool for understanding diurnal glycemic regulation and effects of treatment. Recent studies have used CGM to identify unique glucotypes in individuals with T2DM that can help guide precision treatment (7, 8). Observing nocturnal glucose via CGM may be particularly valuable due to the associations between late-night eating and increased risk of cardiometabolic diseases (9, 10). In one study, late‐night eating under simulated night‐shift conditions disrupted normal glucose rhythms and impaired glucose tolerance, whereas restricting meals to daytime hours preserved glucose tolerance under the same conditions (11). These results highlight the detrimental impact of nighttime eating on glucose regulation and suggest that extending the interval of no caloric intake before sleep may be beneficial. Time-restricted eating (TRE) offers a practical strategy to extend the overnight fasting period and may improve overall glycemic control (12).

## 2 Diagnostics, interventions, and outcomes

**Figure 1:**
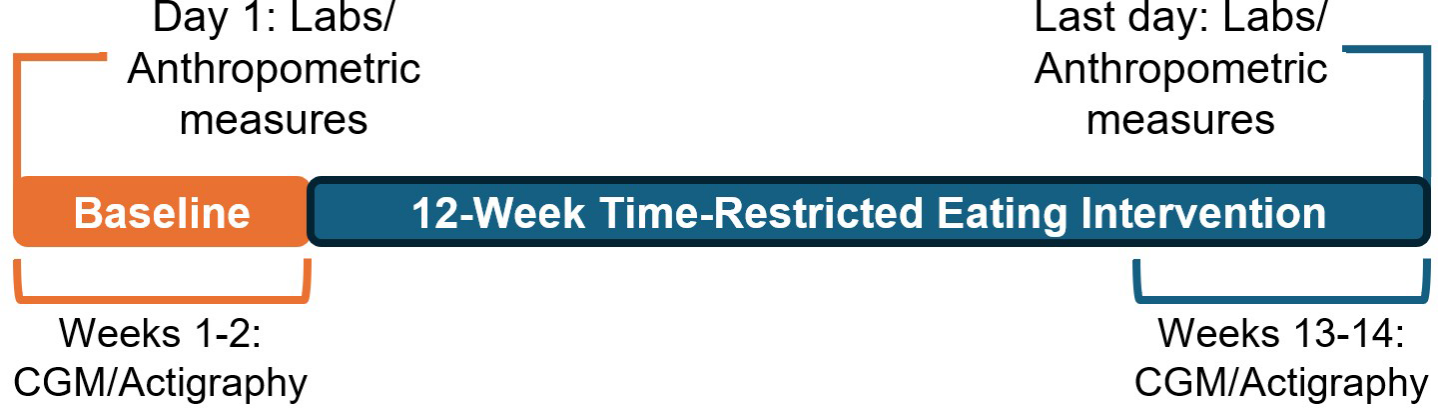
Timeline of patient assessments and intervention. On day 1, labs/anthropometric measurements were taken in the clinic in a fasted state, and patients were given a CGM (Dexcom G6 Pro) and an actigraphy watch (Phillips Respironics Actiwatch Spectrum Plus) to wear for 10 and 14 days, respectively. At the end of week 2, patients returned to the clinic to remove the CGM and actigraphy watch and started the TRE intervention. For the next 12 weeks, patients followed the TRE intervention and had biweekly virtual visits with study staff to receive guidance. At the start of week 13, patients were given a new CGM and an actigraphy watch to wear. On the last day of the study, labs/anthropometric measures were repeated, and the CGM/actigraphy watch was removed.

This case series is from participants in a clinical trial that was conducted at the University of California, San Diego Altman Clinical and Translational Research Institute in La Jolla, California, and includes 3 adults (43, 54, 61 years) with T2DM (NCT05365529). These individuals were identified based on the presence of high nighttime glucose levels (>130mg/dL) at baseline, assessed by CGM. Clinical data, including baseline demographics, diabetes history, body weight, and HbA1c, were collected from medical records and assessments during clinic visits. All 3 patients participated in a clinical research trial to assess cardiometabolic benefits of personalized and consistent 8 to 10-hour TRE aligned to their daytime active phase (must end at least 3 hours before their typical sleep) for 3 months. Participants were provided with standard health advice but were instructed not to change caloric intake or activity level. Eating window and adherence to TRE were assessed with the myCircadianClock smartphone app (13). The eating window was calculated as the mid-95th-percentile time window of all caloric entries within 14 days. Actigraphy watches (Phillips Respironics Actiwatch Spectrum Plus, Murrysville, Pennsylvania) collected 30-second epoch measurements of sleep and activity counts and were worn for 14 days both pre- and post-intervention (**Figure 1**). CGMs (Dexcom G6 Pro, San Diego, CA) measured glucose levels in the interstitial fluid every 5 minutes for 10 consecutive days and were worn both pre- and post-intervention. CGM data were segmented into sleep and wake periods based on actigraphy data. To improve resolution and accuracy, days lacking reliable corresponding sleep data were excluded from analysis. Sleep periods beginning within 3 hours of one another were merged, and any resulting sleep segments shorter than 2 hours were discarded. Sleep periods were only considered valid if they began between 6:01 PM and 8:59 AM. The final set of CGM values corresponding to sleep and wake states was then processed using the rGV package in R (14).

## 3 Case descriptions

**Figure 2:**
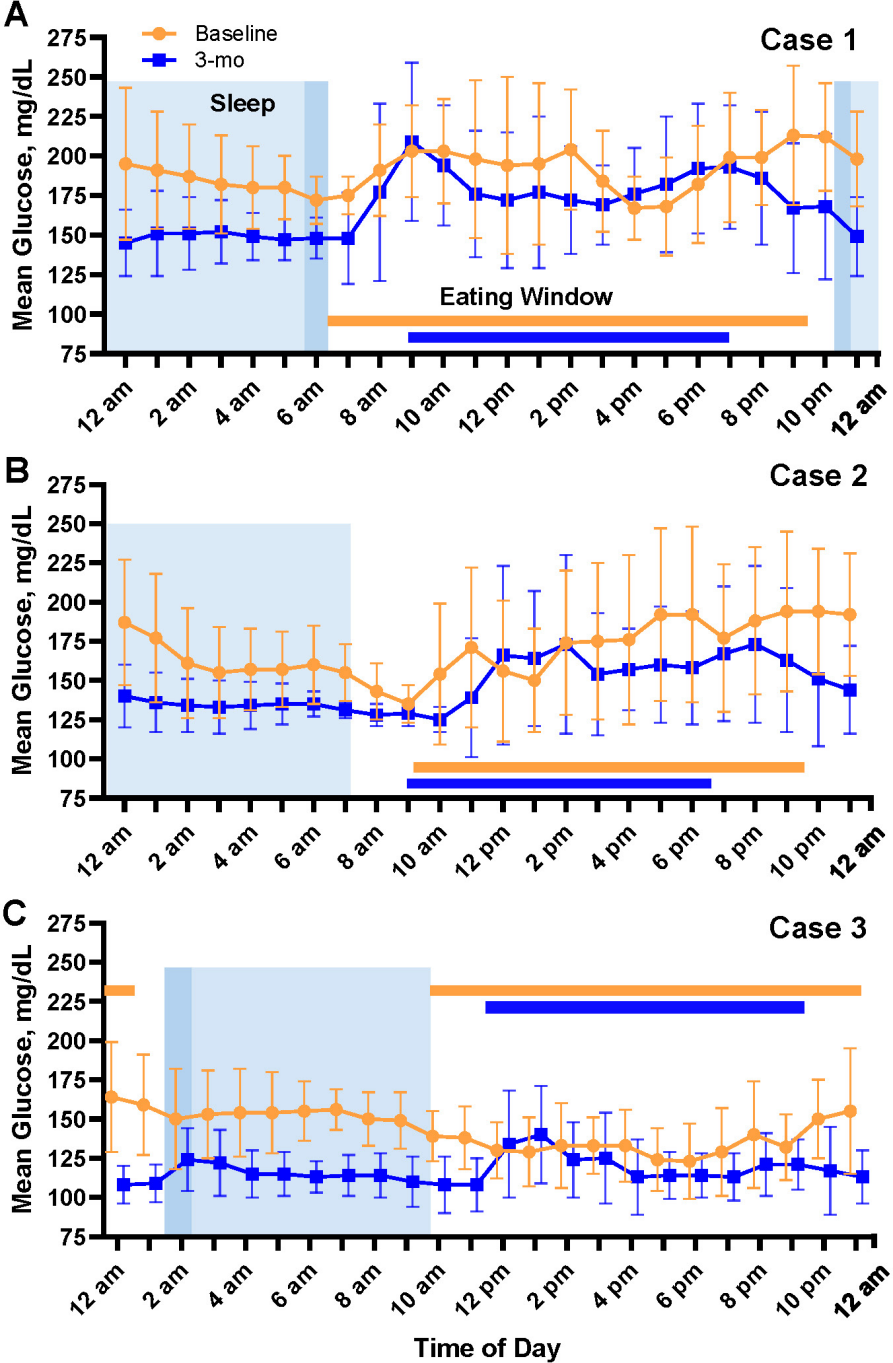
Mean glucose (dots) and SD (error bars) for 10 days of CGM are displayed over a 24-hour day for baseline (orange) and end of the intervention (blue). Cases 1, 2, and 3 are represented as **A, B**, and **C**, respectively. Mean glucose is displayed in hourly bins of samples taken every 5 mins, ex. 12 am represents the mean glucose from 12 am to 1 am over 10 days. Average sleep window indicated for baseline (light blue shaded region) and the last 2 weeks of the intervention (dark blue/overlaid shaded region) based on actigraphy data. The 95th percentile eating interval is shown for baseline (orange bar) and the last 2 weeks of the intervention period (blue bar), including the dates when the CGM and actigraphy devices were worn.

Case 1.A 54-year-old, Black woman with a history of T2DM (diagnosed 13 years before starting the study), obesity (BMI: 30.20), hypertension, dyslipidemia, depression, and anxiety enrolled in the research study while continuing on stable doses of metformin (1000mg BID) and dulaglutide (1.5mg/week) (started medications 4,777 and 714 days, respectively, before starting the study). She presented with an HbA1c of 7.7% and a 15.40-hour eating window (6:16 am - 9:40 pm) at baseline. Her baseline CGM displayed mean glucose and time in range (TIR): (mean (SD), TIR); for wake: 192.86 mg/dL (35.88), 47.38% and sleep: 183.87 mg/dL (27.56), 56.86%. Following 3 months of TRE (target: 10 hours, 9:00 am-7:00 pm), her eating window was shortened to 9.86 hours (9:03 am - 6:55 pm), and her HbA1c decreased from 7.7% to 7.3%. Her 3-month CGM displayed mean glucose and TIR for wake: 177.48 mg/dL (42.81), 60.37% and sleep: 152.13 mg/dL (27.29), 88.83% (**Figure 2A**; **Table 1**). Adherence to her TRE eating window was 93.33% for the 3-month intervention period. Based on actigraphy data, activity counts at the end of the 3-month intervention compared to baseline decreased by 3.73% (-8462.52 arbitrary units (AU).

**Table 1:** Glycemic, demographic, and anthropometric characteristics.

	Case 1	Case 2	Case 3
Demographics, Labs, and Vitals			
Age, y	54	61	43
Sex	Female	Male	Female
Race and/or ethnicity	Black	Asian, Hispanic	Asian, White
Antidiabetic Medications	Metformin (1000mg BID), Dulaglutide (1.5mg/wk)	Semaglutide (0.5mg/wk)	Empagliflozin-Metformin HCl (5-500mg BID), Semaglutide (2mg/wk)
Baseline weight, kg	75.30	112.80	96.90
3-mo weight, kg	71.90	110.30	86.90
Delta weight, kg	-3.40	-2.50	-10.00
Baseline BMI	30.20	33.00	31.80
3-mo BMI	28.80	32.20	28.60
Delta BMI	-1.40	-0.80	-3.20
Baseline HbA1c, %	7.70	7.00	7.00
3-mo HbA1c, %	7.30	6.60	6.20
Delta HbA1c, %	-0.40	-0.40	-0.80
CGM Outcomes			
Baseline mean glucose, mg/dL	190.49 (37.45)	169.75 (44.17)	143.05 (28.04)
3-month mean glucose, mg/dL	168.88 (39.84)	146.98 (35.91)	117.14 (21.31)
Delta mean glucose, mg/dL	-21.61	-22.77	-25.91
Baseline wake mean glucose, mg/dL	192.86 (35.88)	174.17 (51.26)	135.45 (29.69)
3-month wake mean glucose, mg/dL	177.48 (42.81)	149.77 (40.08)	115.61 (20.74)
Delta wake mean glucose, mg/dL	-15.38	-24.40	-19.84
Baseline wake TIR, %	47.38	55.20	89.84
3-month wake TIR, %	60.37	82.71	98.88
Delta wake TIR, %	12.99	27.51	9.04
Baseline sleep mean glucose, mg/dL	183.87 (27.56)	168.84 (36.1)	153.20 (24.7)
3-month sleep mean glucose, mg/dL	152.13 (27.29)	135.51 (16.43)	114.67 (16.18)
Delta sleep mean glucose, mg/dL	-31.74	-33.33	-38.53
Baseline sleep TIR, %	56.86	69.77	84.25
3-month sleep TIR, %	88.83	98.38	99.50
Delta sleep TIR, %	31.97	28.61	15.25
Temporal Eating Patterns			
Baseline 95% eating window, h	15.40	12.25	14.82
3-month 95% eating window, h	9.86	9.50	9.50
Delta 95% eating window, h	-5.54	-2.75	-5.32
Baseline time between last food and bed, h	1.01	2.53	1.65
3-mo time between last food and bed, h	3.49	5.56	4.52
Delta time between last food and bed, h	2.48	3.03	2.87

Mean (SD) shown. Deltas, 3-month Intervention – Baseline. H, hour; Y, years; BMI, body mass index; HbA1c, hemoglobin A1c (glycated hemoglobin); TIR, time in range; TRE, time-restricted eating; CGM, continuous glucose monitoring. 3-mo TRE adherence reported as % of days with no caloric entries outside of the eating window ±15 min buffer, out of total days in study period.

Case 2.A 61-year-old, mixed-race (Hispanic and Asian) man with a history of T2DM (diagnosed 10 years before starting the study), microalbuminuria, obesity (BMI: 33.00), hypertension, dyslipidemia, obstructive sleep apnea on continuous positive airway pressure, and metabolic dysfunction-associated steatotic liver disease enrolled in the research study while continuing on a stable dose of semaglutide (0.5mg/week) (started medication 211 days before starting the study). He presented with an HbA1c of 7.0% and a 12.25-hour eating window (9:15 am - 9:30 pm) at baseline. His baseline CGM displayed mean glucose and TIR for wake: 174.17 mg/dL (51.26), 55.2% and sleep: 168.84 mg/dL (36.1), 69.77%. Following 3 months of TRE (target 9 hours, 10:00 am-7:00 pm), his eating window was shortened to 9.50 hours (9:30 am - 7:00 pm), and his HbA1c decreased from 7.0% to 6.6%. His 3-month CGM displayed mean glucose and TIR for wake: 149.77 mg/dL (40.08), 82.71% and sleep: 135.51 mg/dL (16.43), 98.38% (**Figure 2B**; **Table 1**). Adherence to his TRE eating window was 87.63% for the 3-month intervention period. His actigraphy data showed a 64.58% increase (142382.36 AU) in activity counts at the end of the 3-month intervention compared to baseline. This notable increase in activity may have been influenced by a local camping and hiking trip the participant took during the 2-week assessment period, which was likely not representative of activity throughout the intervention.

Case 3.A 43-year-old, mixed race (White and Asian) woman with a history of T2DM (diagnosed 6 years before starting the study), obesity (BMI: 31.80), polycystic ovarian syndrome, dyslipidemia, hypertension, hypothyroidism, anxiety and depression enrolled in the research study while continuing on stable doses of empagliflozin-metformin HCl (5-500mg BID) and semaglutide (2mg/week) (started medications 98 and 1,178 days, respectively, before starting the study). She presented with an HbA1c of 7.0% and a 14.82-hour eating window (9:48 am - 12:37 am) at baseline. Her baseline CGM displayed mean glucose and TIR for wake: 135.45 mg/dL (29.69), 89.84% and sleep: 153.2 mg/dL (24.70), 84.25%. Following 3 months of TRE (target 9.5 hours, 11:30 am-9:00 pm), her eating window was shortened to 9.50 hours (11:30 am - 9:00 pm) and her HbA1c decreased from 7.0% to 6.2%. Her 3-month CGM displayed mean glucose and time in range for wake: 115.61 mg/dL (20.74), 98.88% and sleep: 114.67 mg/dL (16.18), 99.5% (**Figure 2C**; **Table 1**). Adherence to her TRE eating window was 93.33% for the 3-month intervention period. Activity counts for the actigraphy watches increased by 17.20% increase (25125.32 AU) at the end of the 3-month intervention compared to baseline.

## 4 Discussion

This case series highlights the potential of TRE to improve glucose regulation in individuals living with T2DM on stable GLP-1RA. In these 3 cases, patients with high glucose levels during sleep (>130mg/dL) at baseline (15) had large decreases in glucose levels during sleep, paralleled by reductions in HbA1c. The circadian system orchestrates endogenous rhythms that recur approximately every 24 hours. Circadian regulation of glucose tolerance produces diurnal variation, with tolerance reduced in the evening relative to the morning due to reduced β-cell responsiveness and lower insulin sensitivity later in the day (16), in part due to elevated nocturnal melatonin levels suppressing insulin secretion (17, 18). Late-night eating has been associated with an increased risk of obesity and T2DM (9, 19) and has been shown to lead to elevated glucose levels throughout sleep (11). For individuals with type 2 diabetes, circadian disruption due to mistimed eating may be especially detrimental. Those who consume more than 25% of their daily energy intake after the typical dinner time have been shown to have higher HbA1c levels and an increased risk of diabetes-related complications (20, 21). TRE offers an accessible intervention that intentionally aligns eating to the active phase. This alignment allows melatonin levels to decrease before breakfast, supporting proper insulin response, while also preventing late-night eating to allow glucose levels to fall before sleep.

Additionally, this case series demonstrates the utilization of CGM as a powerful tool for identifying glucotypes that may elicit a specific response to TRE. CGM provides insight into the fluctuation of glycemic patterns across the sleep-wake cycle, allowing for a more targeted therapeutic approach based on an individual’s unique glycemic profile. In conventional medicine, patients are often given standardized recommendations – and while some respond well to these guidelines, others see little benefit despite strict adherence. One size does not fit all, indicating the need for a more personalized approach to medicine. CGMs help fill a critical gap left by traditional glucose monitoring methods, which offer little insight into glucose patterns during sleep. By capturing nocturnal glucose levels, CGMs open a new window for more personalized interventions.

If left unaddressed, elevated nocturnal glucose may increase the likelihood of developing T2DM and related complications. Growing evidence indicates a bidirectional relationship between sleep, diet, and the development of metabolic disorders, including T2DM, and suggests that nighttime postprandial metabolism may have important impacts on sleep quality and overall health (22). In a cross-sectional study of adults with T2DM, participants with high nighttime glucose levels (≥165 mg/dL) had a higher degree of cardiovascular disease markers, independent of daytime glucose levels (23). Monitoring glucose levels during sleep offers a novel, underutilized avenue for targeted interventions to reduce cardiometabolic risk.

In all 3 cases in this series, we observe improvements in mean glucose levels and TIR during sleep after following an 8 to 10-hour TRE intervention aligned with the active phase. These data do not indicate that only individuals with high nocturnal glucose will derive benefits from following TRE, but rather serve to illustrate a specific phenomenon that may apply to individuals with T2DM who portray this glucotype.

We observe a range of benefits (differing decreases in HbA1c, weight, glucose levels, etc.) among the 3 cases presented in this series. Regardless of baseline glucose levels, medication regimen, or activity level, all participants demonstrated improvements in glucose regulation following the 3-month TRE intervention, suggesting that these metabolic changes cannot be attributed to any single co-occurring factor. The additional glycemic benefits derived from implementing TRE on top of standard of care medical therapy, including powerful pharmacological agents like GLP-1RA, highlight the importance of integrating lifestyle interventions in conjunction with medications.

While our findings suggest potential benefits of TRE in glucotypes characterized by high glucose levels during sleep relative to wake, any conclusions made must be considered within the context of the limitations of this case study. The small sample size of 3 patients and the timeline of 3 months prevent generalizability of the results. Longer and larger trials are needed to evaluate the long-term impact of TRE on glycemic control. Due to differing baseline characteristics and medication regimens amongst the individuals presented in this series, it is difficult to isolate the effects of TRE alone. Multiple factors can affect the health outcomes observed in this study, necessitating future studies to better understand the interaction between TRE and various medication classes and to explore whether specific glucotypes may benefit from TRE more than others. Larger, longer-term studies are warranted to further investigate the benefits of TRE across diverse glucotypes and to clarify its interaction with various medication regimens.

## 5 Conclusion

TRE presents an accessible lifestyle intervention that can complement pharmacological therapy to promote cardiometabolic health in individuals living with T2DM. By reducing late-night eating and aligning food intake to support circadian physiology, TRE may help improve nocturnal glycemic regulation. This case series highlights a specific glucotype within T2DM – characterized by elevated glucose levels during sleep – that may be particularly responsive to TRE. These findings underscore the potential of TRE, in combination with CGM, to enable more personalized and effective diabetes management.

## 6 Patient perspectives

Case 1. The patient participated in the clinical trial to improve her overall health. She initially expected that the TRE intervention would be easy to adopt, but found it challenging at times to stay within her self-selected eating window. Barriers to adherence included balancing meal preparation with her work schedule, coordinating mealtimes with family, managing caregiving responsibilities, and delaying morning caffeine intake. Despite these challenges, she felt that the benefits of participation outweighed the time and effort required. She strongly agreed that the study helped her learn more about her health and well-being and reported improvements in glucose levels, digestion, and weight. Overall, she described a positive experience, expressed willingness to recommend TRE to others, and plans to continue practicing TRE with an earlier start time for her eating window.

Case 2. The patient participated in the clinical trial to improve his overall health and hoped to see improvements in blood glucose, cholesterol levels, energy, and weight. He initially expected that the TRE intervention would be difficult to adopt and found this to be the case. Barriers to adherence included hunger, balancing meal preparation with his work and exercise schedule, coordinating mealtimes with family, and managing caregiving responsibilities. Despite these challenges, he felt that the benefits of participation outweighed the time and effort required. He strongly agreed that the study helped him learn more about his health and well-being and reported improvements in energy levels and weight. Overall, he described a positive experience, expressed willingness to recommend TRE to others, and plans to continue practicing TRE with an earlier start time for his eating window.

Case 3. The patient participated in the clinical trial to improve her overall health and hoped to see reductions in weight and HbA1c levels. She initially expected that the TRE intervention would be manageable and found it easier than anticipated, with positive results that exceeded her expectations. Barriers to adherence included hunger, balancing meal preparation with her work schedule, coordinating mealtimes with family, and delaying morning caffeine intake. Despite these challenges, she felt that the benefits of participation outweighed the time and effort required. She strongly agreed that the study helped her learn more about her health and well-being and reported improvements in energy levels, along with reductions in abdominal bloating and joint pain. Overall, she described a positive experience, expressed willingness to recommend TRE to others, and plans to continue practicing TRE with the same eating window.

The patient presented in Case 2 experienced hunger, an expected adverse event. All three participants tolerated the intervention well, with no other adverse or unanticipated events reported.

## Data Availability

The raw data supporting the conclusions of this article will be made available by the authors, without undue reservation.
